# Policy coherence between food security, disaster risk reduction and climate change adaptation in South Africa: A summative content analysis approach

**DOI:** 10.4102/jamba.v14i1.1173

**Published:** 2022-02-22

**Authors:** Annegrace Zembe, Livhuwani D. Nemakonde, Paul Chipangura

**Affiliations:** 1 Unit for Environmental Sciences and Management (African Centre for Disaster Studies), Faculty of Natural and Agricultural Sciences, North-West University, Potchefstroom, South Africa; 2 Disaster Management, Institute of Development Studies, National University of Science and Technology, Bulawayo, Zimbabwe

**Keywords:** climate change adaptation, disaster risk reduction, food security, policy coherence, legislations

## Abstract

Climate change through extreme weather events threatens food security (FS) and the eradication of poverty. Thus, improving FS will require adapting to the impacts of climate change as well as reducing the risks of disasters. However, the nexus between FS, disaster risk reduction (DRR) and climate change adaptation (CCA) is not always reflected in policies, resulting in fragmented implementation. The purpose of this article is to evaluate if there is coherence in the policies for FS, DRR and CCA in South Africa. A qualitative research design was applied, and data were collected through a summative content analysis on 34 policy and legislative documents and 24 key informant interviews (KII). The study found that there are still incoherencies between the current main policy and legislative documents that address CCA, DRR and FS. This study recommends a review of old policy and legislative frameworks promulgated in the 1990s to incorporate cross-cutting issues such as DRR, CCA and FS. This will enhance and strengthen synergies and interconnections between the three policy areas.

## Introduction

Globally, extreme climate and weather events resulting from climate change and variability are on the rise (EMDAT 2021) and are posing an extremely high and increasing disaster risk and food security (FS) challenges (FAO 2021). In most developing countries (where economies are largely agro-based), the recurrence of hydro-meteorological hazards, such as droughts, floods and cyclones, presents FS concerns as they affect agricultural production (Madurapperuma et al. 2020). Food Agriculture Organisation (FAO) (2002) describes FS as:

[*A*] situation that exists when all people, at all times, have physical, social and economic access to sufficient, safe and nutritious food that meets their dietary needs and food preferences for an active and healthy life. (p. 49)

To meet the growing demand for FS under increasingly difficult climatic conditions, efforts to improve FS must consider the nexus between climate change adaptation (CCA), disaster risk reduction (DRR) and FS (Habiba et al. 2016). In this light, there have been significant efforts globally to strengthen coherence between DRR, CCA and FS frameworks to build societal resilience to climate change risks and food insecurity, for example, the 2030 Agenda for Sustainable Development Goals (SDG 2) (United Nations [UN] 2015), the Sendai Framework for Disaster Risk Reduction 2015–2030 (paragraph 19 (h) and 30 (j)) (United Nations International Strategy for Disaster Reduction [UNISDR] 2015) and the Paris Agreement on Climate Change (Article 7.1, 8.1 & 8.4) (United Nations Framework Convention on Climate Change [UNFCCC] 2015).

Policy coherence can be described as an attribute of policy that systematically reduces conflicts and promotes synergies between and within different policy areas to achieve the outcomes associated with jointly agreed policy objectives (Nilsson et al. 2012:396). Building climate resilience in the food sector therefore requires bringing policy coherence to CCA and DRR and management. This helps in bridging the gaps between sectoral organisations for FS, DRR and CCA to share timely and relevant information concerning risks and their management (Habiba et al. 2016). In South Africa, research that supports policy coherence has focused on the alignment of policy objectives (Thow et al. 2018), the need for dialogue (Boatemma, Drimie & Pereira 2018) and integrated institutional arrangements (Pereira & Drimie 2016) as main apparatus needed to foster policy coherence. While there have been some efforts to foster policy coherence between DRR, CCA and FS, most frameworks, models and themes have fallen short of focusing on written content in policy and legislations to show the extent to which use of explicit words could create visible synergies that could easily be identified and used by practitioners from various disciplines.

Against this backdrop, this article seeks to evaluate if there is coherence in the policies for FS, DRR and CCA in South Africa. This will be done by exploring the use of content within CCA, DRR and FS policy and legislative documents with the purpose of understanding the meaning and context in which it is used to promote coherence. Understanding written content (text) in policy coherence is crucial for making replicable and valid inferences from data to their context, with the purpose of providing knowledge, new insights, a representation of facts and a practical guide to action (Elo & Kyngas 2008). After this introduction, the article explores the interlinkages between FS, DRR and CCA. This is then followed by an outline of the methodology used in the study. The findings of the study are then presented and discussed before conclusions are drawn and recommendations made.

## Exploring interlinkages between climate change adaptation, disaster risk reduction and food security policy areas

Food security is expected (if not already doing so) to face increasing challenges from climatic risks that are exacerbated by climate change, especially in the developing world (Balaghi et al. 2010). Meanwhile, the relationship between climate change and disasters has become vividly clear. It is well documented that climate change is changing the frequency, intensity and duration of some disasters, particularly those of hydrometeorological origin. The IPCC (2021) states that human-induced climate change is already affecting many weather and climate extremes in every region across the globe. In summary, climate change is a key source of disaster risk (Cubie & Natoli 2022).

**FIGURE 1:**
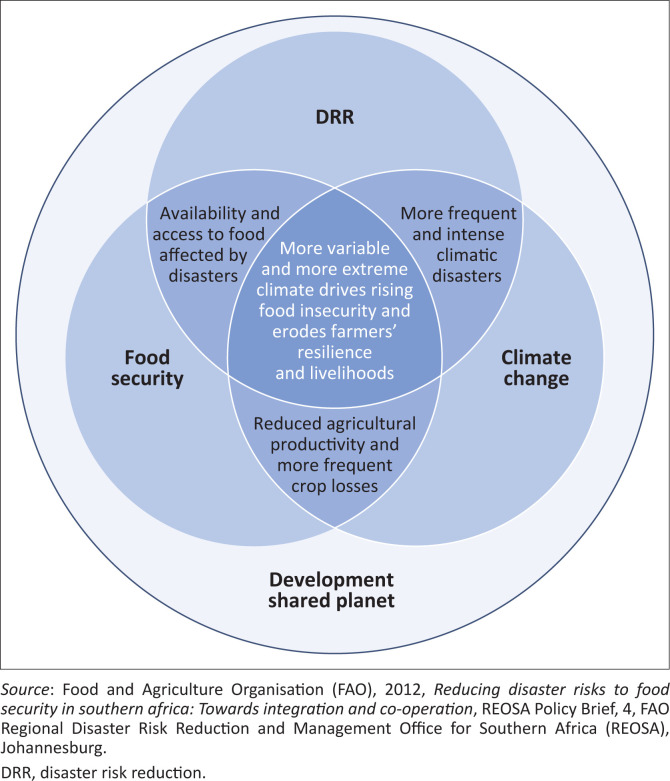
Disaster risk reduction, climate change adaptation and food security nexus.

Given the interlinkages that exist between climate change and disasters and the impacts they have on FS, it is imperative to identify and act on interlinkages that exist between FS, DRR and CCA. However, the interlinkages between the three are only emerging and continuously evolving, with the nexus between them not yet clear nor well documented. As a result, the interlinkages are poorly reflected in policies, resulting in a lack of shared purpose and fragmented implementation (Habiba et al. 2016). This article heavily draws from the work of Habiba et al. (2016) and FAO (2012) who developed a framework depicting the nexus between FS, DRR and CCA (see Figure 1). The framework explores the CC-DRR nexus, DRR-FS nexus, CC-FS nexus and CC-FS-DRR nexus in the centre of the circles. The literature on the complex interlinkages between CC and disaster risk abounds (Becker et al. 2013; Nemakonde & Van Niekerk 2017; Nemakonde et al. 2021). Simply put, CC is the trigger for extreme events (Madurapperuma et al. 2020). These interlinkages have made the integration of the measures to reduce the risk of disasters (DRR) and adapt to the impacts of climate change (CCA) a priority.

Climate change adaptation and DRR share a common conceptual understanding of the components of risk and the processes of building resilience (Turnbull et al. 2013). Both have conceptual synergies of understanding, monitoring and reducing exposure to hazards, addressing vulnerability, raising societal capacities to mitigate and manage risks, and enhancing coping and adaptive capacity to build community resilience (Rani et al. 2020; Sushchenko & Schwarze 2020). Madurapperuma et al. (2020) point out that CCA, coupled with upscaled DRR, contributes to reducing the negative impacts of extreme events and other socio-economic shocks. Because of the shared objectives, the literature (Kelman et al. 2017; Mitchell & Van Aalts 2008) has suggested the integration/alignment/mainstreaming or bringing coherence to the policy areas. Whereas these words have different meanings, they are used interchangeably to denote the bringing together of the different areas.

The nexus between DRR and FS is centred on the premise that disasters affect food availability and access (Masipa 2017). Disastrous events such as floods, tropical cyclones and other extreme events cause damages and losses to the agriculture infrastructure, assets, crops, inputs and productivity (Madurapperuma et al. 2020) which, in turn, impact food safety, quality, and food and nutrition security (FAO 2013). Moreover, disasters breed poverty which, in turn, increases the prevalence of food insecurity and malnutrition (Tirivangasi 2018). Conversely, DRR is a prerequisite and strongly interconnected with alleviating poverty and hunger (FAO 2013). The CC–FS nexus is centred on the role that climate change has on agricultural production and thus FS. According to FAO (2012), food production is declining and the number of impoverished farmers and their dependents is sinking into chronic food insecurity, which is increasing because of increasing risks of unpredictable rainfall and climate-related disasters. As FAO (2008) indicates, climate change affects all four dimensions of FS. Balaghi et al. (2010) share similar views and state that the four dimensions of FS are climate dependent.

As reflected in the introduction of this article, the three policy areas are connected through the variable and extreme climate events that have direct and indirect effects on food insecurity and eroded the farmers’ resilience and livelihoods. In the middle where all three intersect, the overall impact on agriculture-based livelihoods becomes clear and shows the need for an integrated and long-term building of resilience, which could save millions of livelihoods (Habiba et al. 2016). FAO (2012) argues that the nexus between climate variability and change, climate-related disasters, FS and agriculture must be understood and fed into policy development and alignment.

It should, however, be noted that all three issues have component drivers that do not intersect with one another: food insecurity (especially issues of access to food) is driven by numerous socio-economic factors and shocks to the food system; CC has causes and impacts unrelated to DRR and FS; and DRR also includes non-climatic disasters, such as earthquakes and tsunamis (Habiba et al. 2016). Furthermore, FS, DRR and CCA are characterised by different sets of actors at global, regional, national and subnational levels that operate in silos and remain ignorant of the nature and importance of the nexus that exists (Habiba et al. 2016). Besides, the framework presented in this article has revealed strong interdependencies and nexus between FS, DRR and CCA and therefore nations can ill afford to continue implementing them in isolation. According to Madurapperuma et al. (2020), addressing the challenge of food insecurity as a result of an increase in climate variability and extremes and disasters requires coherent policies, strategies and programmes. Whereas it might not be sufficient on its own (Cubie & Natoli 2022), the promotion of coherence is a worthwhile endeavour.

## Methods and procedure

In this study, a mixed methods research design was applied with data collected through a comprehensive literature review, content analysis (CA) of policy and legislative documents relating to DRR, CCA and FS in South Africa and key informant interviews (KIIs). Content analysis was used to identify and quantify content from 34 purposively selected policy and legislative documents to understand the contextual use in relation to coherence (Hsieh & Shannon 2005). Furthermore, thematic analysis was used (Terry et al. 2017:17) to present and analyse data collected from CA and the 24 purposively selected key informants from eight institutions (government sectors and government supported research institutions) during the period September 2019 – December 2019.

### Content analysis of policy document

Content analysis describes a family of approaches for systematic examination of text to identify themes, intents and patterns (Hall & Steiner 2020). In this study, both conventional CA and summative CA were conducted. Conventional CA is an inductive approach which focuses on text data, while summative CA involves counting and comparisons of keywords or content, followed by the interpretation of the underlying context (Hsieh & Shannon 2005). Drawing from Hall and Steiner (2020), the approach for CA in this study involves four stages:

- Searching for and gathering policy and legislative documents from government websites
- Development of keywords and identification of preliminary themes through qualitative inductive reading of text
- Using Atlas.ti to describe policy quantitative attribute and trends using keywords
- Evaluating the themes by qualitative deductive comparison with KII findings.

#### Gathering of national policy and legislative documents

Various policy and legislative documents that address CCA, DRR and FS were identified through searching on government departmental websites. The inclusion criteria for selection were that the following: (1) post-democratic legislation (legislations that dates back from 1994 and are currently used), (2) legislations formulated and published by the South African government, (3) legislations that directly address CCA, DRR and/or FS and (4) other relevant policy documents cited in the legislative documents under review. From the websites, under the legislation section, bills, acts, policies, white and green papers, strategies, and action plans were retrieved and sorted under three categories (CCA, DRR and FS). A total of 34 documents were identified (CCA-12; DRR-8; FS-14).

#### Content analysis of policy and legislative documents

Several steps were followed when analysing the contents of the policy and legislative documents. Initially, a list of key words was developed to allow standardised analysis. The keywords are derived from the focus and interest of study and included CCA, DRR, FS, coherence, integration, climate resilience, disaster risk management, disaster risk, emergency response, food access, food availability, food production, FS and nutrition, sustainability, environment, environmentally friendly, adaptation, and conservation.

The analysis began with searches for occurrences of the identified keywords, counting the number of times that each keyword is mentioned from the identified documents using Atlas.ti. Atlas.ti was used because it can cope with multiple and overlapping codes without losing the context, enabling the researcher to associate codes with chunks of text, patterns to construct classification of codes that reflect the three distinct themes of the analysis (Lewis 2004; Smit 2002). Atlas.ti auto-generated codes for each document using the code ‘D’ (see Table 1). Furthermore, from the documents loaded on Atlas.ti, codes were generated both inductively and deductively, and were grouped into three code clusters (CCA, DRR and FS). The coding system used reflected the code group, code name and the documents it was extracted from, for example, CCA (code group)-adaptation (code name)-FS (documents extracted from). This process was mainly done to quantify the occurrence of every code in each document. For the coherence variable, the code group was shown followed by code name (CCA-coherence). Lastly, the documents where the keywords are mentioned were thoroughly read and text analysed to verify the context in which the word is used.

**TABLE 1:** List of climate change adaptation, disaster risk reduction and food security documents in South Africa analysed.

Codes	Document name
D1	White Paper Marine Fisheries Policy for South Africa, 1997
D2	National Climate Change Response White Paper, 2011
D3	National Climate Change Response Green Paper 2010_ Department of Environmental Affairs briefing _ PMG
D4	National Climate Change Adaptation Strategy, 2019
D5	*National Environment Management Act*-107-of-1998 amended 2013
D6	National Climate Change Response Strategy South Africa, 2004
D7	*National Water Act* 36, 1998
D8	*National Environmental Management Act*, 1998 (107): Regulations: Environmental Management Framework, 2010
D9	*National Environment Management Act Air Quality Act* of 39, 2004
D10	*South African Weather Services Act* 8, 2001
D11	Climate Change Bill, 2018
D12	*National Forest Act*, 1998
D13	Disaster Management Monitoring and Evaluation Framework, 2014
D14	National Disaster Risk Management Education and Training Framework, 2013
D15	*Disaster Management Amendment Act*, 2015
D16	Disaster Management Urban Search and Rescue Framework, 2014
D17	Disaster risk Management White Paper, 1999
D18	Disaster Risk Management Green Paper, 1998
D19	Disaster Risk Management Policy Framework, 2005
D20	*Disaster Risk Management Act* 57, 2002
D21	South Africa Agricultural Policy Action Plan, 2014
D22	Gazette-Climate Smart Agriculture Strategy Framework, 2018
D23	Draft Conservation Agriculture Policy, 2017
D24	Department of Agriculture Forestry and Fisheries draft sector IGDP, 2010
D25	Policy on Agriculture in Sustainable Development – 8th Draft
D26	Indigenous Knowledge Systems Strategy, 2008
D27	Rural Development Framework Policy, 2013
D28	Draft Climate Change Sector Plan for Agriculture, 2015
D29	Green Paper Land Reform, 2011
D30	National Policy for Food and Nutrition Security, 2014
D31	Integrated Food Security Strategy, 2002
D32	Mechanization Support Policy Framework, 2014
D33	Fetsa Tlala – Integrative Food Production Initiative, 2013
D34	Household Food and Nutrition Strategy for South Africa, 2013

### Key informant Interviews

In this study, purposive sampling was applied to select respondents. Purposive sampling is a form of sampling that selects participants because they are well informed on and understand the research problem (Creswell 2007). A total of 24 key informants from eight institutions including Department of Agriculture Land Reform and Rural Development (DALRRD), Department of Environment, Forestry and Fisheries (DEFF) (Climate Change Directorate), Department of Cooperative Governance and Traditional Affairs (National Disaster Management Centre [NDMC]), Agricultural Research Council (ARC), Human Sciences Research Council (HSRC), Water Research Commission (WRC), South African Weather Services (SAWS), and Agri South Africa (AGRISA) participated in the study. Respondents included three senior managers, that is, Chief Directors, Directors and Deputy Directors from each of the eight institutions.

Data were collected through face-to-face interviews, telephonic interviews, virtual or online platforms and emails, allowing participants to choose a platform convenient to them. Interview sessions were recorded with consent granted by the interviewees. The collected data were transcribed verbatim and were analysed and presented using thematic analysis. The analysis followed the six-phase process established by Terry et al. (2017:23–24). The process began with (1) familiarising with the data (browsing through data to get the meaning). Phase 2 and 3 (generating and constructing codes), respectively, were skipped as it was out of scope of the study. Phase 4 and 5 (reviewing potential themes and defining and naming themes) wherein themes that emerged from CA were used to allow for proper comparison of the findings. The themes that emerged from the study are reflected in Table 2.

**TABLE 2:** Themes that emerged from the study.

Themes	Sub-themes
Interlinkages between DRR, CCA and FS	Interlinkages between CCA and DRR
	Interlinkages between CCA and FS
	Interlinkages between FS and DRR
The extent to which policy and legislative content promote policy coherence	Content in CCA policies and legislation promoting coherence
	Content in DRR policies and legislations promoting coherence
	Content in FS policy and legislation promoting coherence
Possible challenges and opportunities for not/or using explicit content to promote coherence	Challenges and opportunities for coherence between FS and CCA policies
	Challenges and opportunities for coherence between FS and DRR policies
	Challenges and opportunities for coherences between DRR and CCA policies

CCA, climate change adaptation; DRR, disaster risk reduction; FS, food security.

## Findings and discussions

This section presents and discusses the findings from Summative content analysis (SCA) and KIIs on policy coherence between CCA, DRR and FS in South Africa. The presentation focuses on the use of content within CCA, DRR and FS policy and legislations in order to understand the meaning and context in which it is used to promote coherence. The section begins by presenting the interlinkages between DRR, CCA and FS policy and legislations. The subsequent sub-sections present findings on the extent to which DRR, CCA and FS content promote policy coherence and possible challenges and opportunities for not/or using explicit content to promote coherence. Attention is given to the extent in which use of explicit words creates visible synergies that could easily be identified and used by practitioners from various disciplines.

### Interlinkages between disaster risk reduction, climate change adaptation and food security policy and legislations in South Africa

As highlighted above, efforts to improve FS under increasingly changing climatic conditions require policies from different sectors that influence FS to be interlinked. In this section, findings on the interlinkages between DRR, CCA and FS policy and legislations in South Africa are presented. The findings are presented under three sub-sections, namely, (1) interlinkages between CCA and FS, (2) interlinkages between CCA and DRR and (3) interlinkages between FS and DRR. The presentation mainly focuses on identifying keywords that reflect interlinkages between the three policy areas and how KIIs converge or diverge to SCA findings.

#### Interlinkages between climate change adaptation and disaster risk reduction policy and legislations

Meeting the demand for FS in increasingly extreme climate and weather events will undoubtedly require bringing policy coherence between CCA and DRR. This is because CCA and DRR are connected through the overarching purpose of reducing losses because of climate-related hazards and building communities’ resilience against disasters. In this study, the interlinkages between CCA and DRR policy and legislations are summarised in Table 2. As shown in Table 3, only two CCA/DRR codes were found in DRR documents compared to five DRR/CCA codes found in CCA documents. No quotations were found under the codes ‘CCA-climate resilience-DRR’ and ‘CCA-environmentally friendly-DRR’. This implies that these codes are operating in silo, which may complicate efforts to build resilience. Interlinkages were only found in codes ‘CCA-sustainability-DRR’ (four quotations) and ‘CCA-climate change adaptation-DRR’ (five quotations). The *Disaster Management Amendment Act* (D15) had a total of five CCA quotations that referred to the need to engage DEFF when responding to climate and disaster risks in South Africa. D13/14/18/19 had each one quotation that recognises climate change as a concept that exacerbates disaster risk if not managed well. However, these codes did not convey the content in clarity on which action to be taken to work in harmony. This lack of clarity may compromise communication between legislations and implementation of programmes because CCA and DRR have complimenting policy goals that need to be addressed holistically (Lei 2014; Mercer 2010).

**TABLE 3:** Interlinkages between climate change adaptation and disaster risk reduction policy and legislations.

CCA and DRR odes	No. of times codes were mentioned	Total no. of codes
CCA-climate resilience-DRR	0	0
CCA-environmentally friendly-DRR	0	0
CCA-sustainability-DRR	D13 – 1; D18 – 1; D14 – 1; D19 – 1	4
CCA-climate change adaptation-DRR	D15 – 5	5
DRR-disaster resilience-CCA	0	0
DRR-disaster risk reduction-CCA	D2 – 4; D4 – 7	11
DRR-disaster risk-CCA	D2 – 8; D4 – 12; D11 – 1	21
DRR-disaster risk management-CCA	D2 – 2; D4 – 2	4
DRR-emergency response-CCA	D2 – 1; D4 – 4	5
DRR-rehabilitation-CCA	D2 – 1; D5 – 7; D6 – 1; D7 – 6	16

*Source:* Derived from Smit, B., 2002, ‘Atlas.ti for qualitative data analysis’, *South African Journal of Psychology* 20(3), 65–76.

CCA, climate change adaptation; DRR, disaster risk reduction.

Interestingly, almost all the DRR/CCA codes had some connection to DRR issues as shown in Table 2 – DRR-disaster risk-CCA (21 quotations), DRR-rehabilitation-CCA (16 quotations), DRR-disaster risk reduction-CCA (11 quotations), DRR-disaster risk management-CCA (four quotations), DRR-emergency response-CCA (five quotations). Disaster risk reduction/CCA codes particularly D2/4 were used to enhance short-term CCA and planning strategies towards early warning systems and forecasting. Notably, DRR-disaster risk management-CCA code was used as a reference for developing disaster management plans, while DRR-emergency response-CCA code was used as a need for managing inevitable climate change impacts to build environmental resilience. However, the fact that most DRR/CCA codes were found in 2 (D2/4) out of 12 documents shows that other CCA documents are treating DRR issues in silo, which hinder policy coherence. Only documents (D5/6/7) formulated in the 1990s used the DRR-rehabilitation-CCA code and the context it was used did not reflect the need for coherence.

#### Interlinkages between climate change adaptation and food security policy and legislations

In terms of the interlinkages between CCA and FS policy and legislations, findings from SCA (see Table 4) indicate that all FS/CCA codes analysed for this study and CCA issues are well interlinked to FS. The code ‘CCA-climate change-FS’ had the highest number (476) of CCA quotations in FS policy and legislation documents followed by the code ‘CCA-adaptation-FS’ with 258 quotations. These results are not surprising given that the term ‘climate change’ is used to portray threats to the food sector and is a variable that should be addressed to reduce its impact on FS. Most FS documents developed before the year 2000 preferred to use traditional terms such as conservation, sustainability, and environment when referring to climate change risks. After the year 2000, the FS documents started to recognise climate change as an interlinked variable to FS. Codes such as ‘CCA-climate change adaptation-FS’ and ‘CCA-adaptation-FS’ scored many quotations in documents that specifically address climate change in DALRRD especially in D28. Mostly, these codes when mentioned referred to the need to work with DEFF to apply CCA measures such as climate smart agriculture (CSA) to improve FS in South Africa.

**TABLE 4:** Interlinkages between climate change adaptation and food security policy and legislations.

CCA and FS codes	No. of times codes were mentioned	Total no. of codes
CCA-climate change adaptation-FS	D21 – 1; D22 – 3; D23 – 1; D28 – 10	15
CCA-adaptation FS	D21 – 6; D22 – 49; D23 – 3; D24 – 12; D25 – 2; D26 – 4; D28 – 182	258
CCA-climate change-FS	D21- 17; D22 – 85; D23 – 6; D24 – 30; D25 – 9; D28 – 317; D30 – 12	476
CCA-climate resilience-FS.	D 22 – 5; D22 – 1	6
CCA-conservation-FS	D21 – 22; D22 – 15; D23 – 35; D24 – 22; D25 – 24; D26 – 9; D28 – 39; D32 – 2; D33 – 2	
CCA-environment-FS	D23 – 18; D24 – 66; D25 – 50; D26 – 13; D28 – 59; D29 – 2; D30 – 4; D31 – 6; D32 – 6; D33 – 2	226
CCA-sustainability-FS	D21 – 14; D22 – 8; D23 – 5; D24 – 26; D25 – 9; D26 – 2; D28 – 13; D32 – 2; D33 – 2	
FS-food availability-CCA	0	0
FS-food access-CCA	0	0
FS-agriculture-CCA	D2 – 27; D3 – 7; D4 – 22; D5 – 1; D6 – 12; D11 – 1	70
FS-food production-CCA	D2 – 3; D3 – 1; D4 – 1	5
FS-food security and nutrition-CCA	0	0
FS-food security-CCA	D2– 5; D3 – 2; D4 – 3; D6 – 3	14

*Source:* Derived from Smit, B., 2002, ‘Atlas.ti for qualitative data analysis’, *South African Journal of Psychology* 20(3), 65–76.

CCA, climate change adaptation; FS, food security.

While in all CCA/FS codes there was good link between FS and CCA, only three FS/CCA codes (FS-agriculture-CCA (70 quotations), FS-food security-CCA (14 quotations) and FS-food production-CCA-(5 quotations)) had some mention of FS in CCA documents, as shown in Table 3. The CCA documents (D2/3/4/5/6) that mentioned FS issues recognise the food sector as the most vulnerable sector to climate risks. Therefore, D2/3/4 emphasised the need to preserve small-scale farmers relying mostly on dry land for food production. The fact that ‘FS-food security and nutrition-CCA’ code did not appear anywhere in the CCA documents showed a lack of focus on the nutrition part of food security in South Africa, which Ruel (2013) argued for further research. Fortunately, the occurrence of ‘FS-food security-CCA’ code in D2/3/4/6 showed an avenue to promote coherence between FS and CCA legislations. The context in which the code appeared reflected how CCA documents acknowledge the impact of climate risks on FS and advocate for the need to use CSA.

#### Interlinkages between food security and disaster risk reduction policy and legislations

The findings on interlinkages between FS and DRR policy and legislations are summarised in Table 5. As shown in Table 4, no quotations were found in the ‘DRR-disaster risk reduction-FS’ code within the FS documents. This shows lack of coherence between DRR and FS legislations given the importance of DRR measures in prevention, response and recovery strategies in the food sector. The remaining DRR keywords (resilience, disaster risk management, disaster risk and emergency response) reflected how the food sector lacks coherence when dealing with climate and disaster risks. This was shown in the context of lack of accessible and reliable information for external engagements and few social platforms to engage civil societies and other relevant stakeholders who advocate for alignment of FS and DRR issues. Arguably, among widely used comprehensive documents such as D30 and D31, no DRR code was recorded. This oversight specifically shows how limited the content in FS legislations is, in terms of lack of direct keywords that help to engage practitioners, sectors and communities who are the end users or implementers of such policies. Such circumstances, according to Campbell et al. (2016) and Lipper et al. (2014), brew a fertile ground for incoherencies, which could prohibit a food secure community. Of significance is D33, whereby, regardless of how sensitive food production is to disaster risks in South Africa, did not use DRR codes as terms of references to align with its activities.

**TABLE 5:** Interlinkages between food security and disaster risk reduction policy and legislations.

DRR and FS codes	No. of times codes were mentioned	Total no. of codes
DRR-disaster resilience-FS	D 28 – 2	2
DRR-disaster risk management-FS	D 22 – 1; D28 – 2	3
DRR-disaster risk reduction-FS	0	0
DRR-disaster risk-FS	D 22 – 2; D28 – 3	5
DRR-rehabilitation-FS	D22 – 2; D23 – 2; D24 – 3; D25 – 3; D28 – 6; D33 – 1	17
DRR-emergency response-FS	D 22 – 2	2
FS-food access-DRR	0	0
FS-conservation-DRR	D 14 -1; D18 – 7	8
FS-food availability-DRR	D18 – 1	1
FS-food production-DRR	0	0
FS-food security and nutrition-DRR	0	0
FS-food security-DRR	D14 – 3; D19 – 6; D18 – 38; D20 – 2	50

*Source:* Derived from Smit, B., 2002, ‘Atlas.ti for qualitative data analysis’, *South African Journal of Psychology* 20(3), 65–76.

DRR, disaster risk reduction; FS, food security.

Findings from SCA, as shown in Table 4, indicate that DRR documents (FS-food security-DRR code in D14/18/19/20) acknowledge the linkages between FS and DRR. For example, D18 shows that, during its formulation stage, officials from DALRRD, DEFF and other role players were consulted, which could be the reason why the content is inclusive and promotes policy coherence. Correspondingly, D19/20 uses National Disaster Management Advisory Forum (NDMAF) and Intergovernmental Committee on Disaster Management (ICDM) to engage all stakeholders including DALRRD in management of floods and droughts to protect FS. The FS-food availability-DRR code that featured in D18 also seeks to engage DALRRD as a key player in early warning systems of staple foods. The remaining three codes (food access, production, FS and nutrition) did not feature anywhere in the DRR documents because they address FS from a broader perspective rather than its dimensions. FS-conservation-DRR code only appeared in D14/18 to encourage water conservation and drought proofing as a DRR measure that helps to improve FS.

Regarding the KIIs findings, majority of respondents described CCA, DRR and FS as interlinked concepts with similar goals in building resilience and adaptive capacity towards the FS. The following statement by a respondent from HSRC captures the sentiments shared by those who feel that the three policy areas are interlinked:

‘CCA, DRR & FS are undoubtedly interlinked and if legislations and technocrats treat them as such, coherence would be inevitable’. (Participant 1, Male, 9 September 2019)

However, respondents from AGRISA, NDMC and Directorate of Climate Change Adaptation (DCCA) argued that the content in some policy and legislations is still influencing a separation between them, which is compromising the achievement of each other’s interlinked goals. For example, one respondent from DALRRD described FS as, ‘an outcome of what institutions that address DRR and CCA did or did not do’. A few respondents from NDMC and DCCA confirmed to share responsibility but said that FS policies should state the responsibilities with clarity to invoke their attention and interests to partake in FS issues not the other way round. Moni et al. (2007) also emphasise the importance of presenting the who, what, when, why and how aspects of content when conveying information to external audiences. One respondent from HSRC said that, currently in areas where interlinkages are witnessed, resources are shared and used efficiently, cross-cutting terms such as ‘resilience’ are being utilised; collective methods, strategies and tools are used to reduce the impact of climate and disaster risk on FS. Finally, the KII findings established that the majority of respondents recognise the interconnectedness of CCA, DRR and FS and in their responses agreed to work on building content that reflects that.

### The extent to which disaster risk reduction, climate change adaptation and food security content promote policy coherence

Under this theme, three sub-themes were established to determine the extent in which content in CCA, DRR and FS policy and legislations is promoting policy coherence. The sub-themes are: (1) content in CCA policy and legislations promoting coherence; (2) content in DRR policy and legislations promoting coherence; and (3) content in FS policy and legislations promoting coherence. Codes used to analyse the sub-themes included coherence, coordination, cooperation, collaboration, integration and mainstreaming.

#### Content in climate change adaptation policies and legislation promoting coherence

Given that climate change is the trigger for extreme events that adversely affect FS, it is important to find out how the content in CCA policies and legislation promotes coherence between CCA, DRR and FS. To determine the extent in which CCA codes were used to promote coherence, various keywords such as coherence, cooperation, and coordination were created. Table 6 summarises the findings on content in CCA policy and legislations that promote coherence. Most content in CCA policies and legislation promoting coherence centred on CCA-coordination (41 quotations) while CCA-integration and CCA-mainstreaming had 29 quotations each. Only three quotations centred on CCA-coherence.

**TABLE 6:** Content in climate change adaptation policies and legislation promoting coherence.

CCA codes	No. of times codes were mentioned	Total no. of codes
CCA-coherence	D2 – 1; D4 – 2	3
CCA-cooperation	D1 – 12; D2 – 6; D4 – 3; D5 – 2; D6 – 5	28
CCA-coordination	D1 – 1; D2 – 15; D3 – 4; D4 – 4; D5 – 2; D6 – 10; D11 – 5	41
CCA-integration	D1 – 2; D2 – 5; D4 – 1; D5 – 3; D6 – 8; D31 – 1	29
CCA-mainstreaming	D 2– 1; D4 – 16; D5 – 2	29

*Source:* Derived from Smit, B., 2002, ‘Atlas.ti for qualitative data analysis’, *South African Journal of Psychology* 20(3), 65–76.

CCA, climate change adaptation.

Among CCA documents analysed, D4 used CCA-integration code to specifically emphasise the need to integrate with DRR and FS legislations. While D1/2/3/5/6/9/11 highlighted the need for coherence between DRR and FS, they did not specifically mention the exact institutions by name but rather described them as government sectors or businesses or civil societies. According to Radhaswamy and Zia (2011), words or information that is not clearly phrased is likely to convey a message that is amiss. In this case, the end users, which are DRR, and FS practitioners and communities, might not understand the brevity of CCA content and might decide not to act coherently. In support, KII findings established that lack of explicitness in content gives no clarity around mandates, risking practitioners to duplicate work with limited funds that could be better used. It was also noted that, some documents specifically D1/2/5/31, when referring to coherence, mostly referred to vertical coherence, which limited horizontal engagements. In addition, the CCA content shows no central platform where CCA-, DRR- and FS-related data are shared, which could be the reason legislations are still using different terms, methods and strategies as opposed to Gero, Méheux and Dominey-Howes (2011) bid for a one-stop-shop for cross-cutting information. Thus, the study draws from D4, and argues that there is lack of coherence or coordination between legislations and institutions that are addressing CCA, DRR and FS policy areas. This is so despite the existence of Intergovernmental Committee on Climate Change (IGCCC) and National Committee on Climate Change (NCCC) platforms identified in CCA documents, which are avenues created to ensure stakeholder participation and new partnerships with relevant parties in an integrated manner.

#### Content in disaster risk reduction policies and legislations promoting coherence

As noted above, disaster risk, climate change and FS are closely connected. Various hazards especially those of hydro-meteorological origisn, propelled by climatic change and variability, pose an extremely high and increasing disaster risks to FS. This connection would require policy coherence between FS, DRR and CCA in order to minimise the devastating impacts of disasters on FS. Table 7 summarises the findings on content in DRR policies and legislation that promote coherence between DRR, CCA and FS. As shown in Table 6, most content in DRR that promote coherence centred mostly on coordination (59 quotations) and integration (53 quotations).

**TABLE 7:** Content in disaster risk reduction policies and legislation promoting coherence.

DRR codes	No. of times codes were mentioned	Total no. of codes
DRR-coherence	0	0
DRR-collaboration	D13 – 1; D14 – 8; D16 – 2; D18 – 3	14
DRR-cooperation	D16 – 5; D18 – 16	21
DRR-coordination	D13 – 8; D14 –3; D16 – 26; D18 – 22	59
DRR-integration	D13 – 1; D14 – 18; D18 – 6; D19 – 16; D20 – 10	53
DRR-mainstreaming	D 14 – 6; D19 – 1	7

*Source:* Derived from Smit, B., 2002, ‘Atlas.ti for qualitative data analysis’, *South African Journal of Psychology* 20(3), 65–76.

DRR, disaster risk reduction.

The frequency in the use of codes that refer to ‘coherence’ in the DRR documents shows that there is an effort to promote policy coherence within the sector. However, among the six codes created to determine the extent to which DRR policy and legislations are promoting coherence, the main keyword ‘coherence’ did not appear in any of the DRR documents. This significant oversight either shows that the concept is still new or evolving as argued by Gero et al. (2011). Briefly, when D13/14/16/18 mentioned DRR-collaboration code, the emphasis was to collaborate with the Weather Bureau, Water Affairs, Department of Education and other relevant stakeholders to improve early warning systems. In like manner, the context in which DRR-cooperation code was used referred to the need for cooperation with relevant stakeholders but did not explicitly mention institutions by name, which in a way reduces the sense of obligation to DRR issues. In addition, the way coordination and integration keywords were used in D6 was to reveal the lack of coordination between DRR and CCA/FS activities. In terms of D16, there is lack of coordination in information dissemination, focal points to provide direction for implementation and lack of systematic set of guidelines and standards that should be adhered to between institutions. Finally, DRR-integration featured in a way that encourages rapid integration of information from different data sources and channels that reaches decision makers promptly. Although, some DRR codes seek to promote coherence using NDMAF and other platforms as cited in D19/20, the extent in which the content is used requires revision in terms of altering ‘quarterly’ to ‘regular’ meetings to strengthen the readiness of external stakeholders whenever an emergency event arises.

#### Content in food security policy and legislation promoting coherence

The nexus between FS and disasters as highlighted above would require that FS policy and legislation recognise that it should be connected to CCA and DRR policies and legislation in order to effectively deal with FS challenges. Table 8 presents the findings on content in FS policies and legislation that promote coherence between FS, DRR, and CCA. As shown in Table 7, most content centred on mainstreaming (66 quotations), coordination (61) quotations, and integration (45 quotations) while only five quotations centred on FS-coherence.

**TABLE 8:** Content in food security policies and legislation promoting coherence.

FS codes	No. of times codes were mentioned	Total no. of codes
FS-coherence	D22 – 1; D23 – 1; D25 – 1; D28 – 1; D29 – 1	5
FS-collaboration	D21 – 5; D22 – 12; D23 – 3; D24 – 6; D26 – 4; D28 – 5; D29 – 1	36
FS-cooperation	D21 – 1; D22 – 2; D25 – 1; D24 – 7; D26 – 5; D28 – 3; D31 – 1	24
FS-coordination	D21 – 6; D22 – 26; D24 – 8; D26 – 2; D28 – 4; D29 – 1; D30 – 1; D31 – 3; D32 – 2; D34 – 8	61
FS-integration	D21 – 9; D22 – 15; D23 – 2; D24 – 4; D25 – 4; D26 – 2; D28 – 5; D29 – 2; D30 – 1; D31 – 1	45
FS-mainstreaming	D 22 – 63; D25 – 63; D28 – 2	66

*Source:* Derived from Smit, B., 2002, ‘Atlas.ti for qualitative data analysis’, *South African Journal of Psychology* 20(3), 65–76.

FS, food security.

The codes created to determine the extent to which FS policy and legislations are promoting coherence reflected a silo mentality towards CCA and DRR. For example, the context in which the coherence keyword was used in D21/23 /28/25/29 showed that there is lack of coherence between FS and DRR/CCA. However, collaboration, coordination and cooperation keywords were used as drivers to engage external agencies to establish integrated approaches to climate change, indigenous knowledge systems and institutional support networks. Twenty-four quotations from D21/22/24/25/26/28/29 showed that weak institutional support networks that relate to disaster management systems and lack of a structured system that deals with FS disasters are always a hindrance to effective coherence. While some documents used the integration code to refer to integration with other government sectors, D30/29/26/28/31/21/22 mainly emphasised for internal integration within the food sector. These documents described the food sector as an entity that lacks integration because of weak governance structures and fragmented content that does not explicitly refer to other FS legislations. Therefore, Boatemma et al. (2018) and Pereira and Drimie (2016) advocated for internal coherence between FS policies first before external alignments with CCA and DRR policies.

However, findings from KIIs have shown two different positions in the way they view the extent to which content is promoting coherence. Majority of respondents from DALRRD, DCCA and NDMC said that the content in their legislations is promoting coherence to a greater extent because they have created vast of policies that address CCA, DRR and FS concepts. On the other hand, few respondents from WRC, ARC, South African Weather Services (SAWS) and HSRC discussed how the creation of vast standalone documents has negatively replaced the use of relevant content that is needed to drive coherence between sectors. One respondent from AGRISA expressed this concern like this:

‘The fact that we have policies that directly address CCA, DRR& FS doesn’t mean the legislations have the right content that promote coherence because … a policy, in itself is not discernible, but the actual written words in the document’. (Participant 15, Male, 27 September 2019)

Although some respondents from DALRRD, DCCA and NDMC gave guidelines and procedures that government sectors follow when formulating policy and legislations in South Africa, one respondent from HSRC argued that, with absolute silence in other documents especially the leading ones, does not reflect the inclusivity and transparency advocated by such guidelines. The same HSRC respondent finally gave a recommendation for the need to focus more on the written content in policies rather than policies themselves.

### Possible challenges and opportunities for not/or using explicit content to promote coherence

Under this theme, three sub-themes were created to present analysis of challenges and opportunities that could be brought by not/or using explicit CCA, DRR and FS terms to promote coherence. These include challenges and opportunities for coherence between FS and DRR policies; challenges and opportunities for coherences between DRR and CCA policies; and challenges and opportunities for coherence between CCA and FS policies.

#### Challenges and opportunities for coherence between food security and climate change adaptation policies

Challenges that have been drawn from D22/25/28 included culture and language barriers whereby people or farmers could be sensitive to certain new words used to drive coherence, which could cause resistance to CSA messages. In support, KIIs findings showed that this could be influenced by people or farmers who are still accustomed to key terms that were used in the 1990s legislations such as conservation farming or rehabilitation practices. Again, the fact that these documents are still functional currently raises a lot of concerns in terms of need for imminent review to create avenues for coherence.

#### Challenges and opportunities for coherence between food security and disaster risk reduction policies

The challenge that emanated from this sub-theme was rooted from the way policy content is designed and the people or institutions that sits at the table during the process. This came about after observation from DRR legislations that do not use the word ‘coherence’ in their content, which in a way complicates understanding of the need for coherence across the FS sector. In the same manner, both SCA and KII findings have shown the challenges of turf problems with DALRRD and FS policies whereby they are establishing policies that address CCA and DRR independently instead of reviewing the content in their policies to integrate terminology that drives coherence with relevant stakeholders. Organisation for Economic Cooperation and Development (2018) referred to such challenges to be caused by political context in which these policies exist whereby politicians are more concerned with distribution of policy benefits during implementation than on broad ends of policies as they are designed. Such a scenario questioned the whole policy-making process in South Africa regarding who have a say in the choice of policy content and recommended it for further research.

#### Challenges and opportunities for coherences between disaster risk reduction and climate change adaptation policies

This sub-theme has shown areas of convergences between SCA and KII findings whereby they indicated opportunities that were brought by using explicit terms in both legislations especially in D15. Majority of respondents from KIIs agreed that content matters because most humans respond and act on things they see. This means that, if nothing is mentioned or seen in the content, no one would know, let alone feel obligated to act. One respondent from NDMC said that:

‘… [*A*]s DRR was evolving since 1994, there was no mention of CCA in any of our legislations … once it was added in DMAA of 2015, it automatically created an opportunity for CCA legislations to cite the Act and align their activities with it’. (Participant 10, Female, 25 September 2019)

While other respondents from DCCA expressed that it is not a matter of mentioning of words or terms but the context in which it is mentioned, a few respondents from NDMC agreed that, although that may be the case, the fact that it is mentioned in a legislation shows a degree of accepting the connection between legislations and that alone can be used as an avenue for policy coherence. Although respondents from HSRC, SAWS and ARC described the use of explicit content as a way needed to clarify roles and responsibilities between sectors, they also cited a possibility of organisational turf problems when it comes to sharing of resources. Finally, this argument brought majority of respondents to recommend the need to incentivise institutions that are promoting coherence.

## Conclusion

Bringing coherence between DRR, CCA and FS policy areas is espoused as one of the strategies to sustain FS. Despite the interlinkages identified between these policy areas, many policies have not yet picked up the button bringing synergy between related policies. Ultimately, addressing the risk and impacts of disasters including climate risks on FS coherently remains a challenge. The findings of this study have revealed that there are incoherencies between the main policies and legislation that address DRR, CCA and FS in South Africa despite some level of synergies in the contents of some policy documents despite some minor differences, there is convergence between the findings of CA and the findings from KII.

This article submits that bringing coherence in the three policy areas will require explicit and precise contents of the policy documents to acknowledge the interlinkages that exist between the policy areas. This will require a review of the policy documents and amendments of the legislative documents. This is because legislation and policy frameworks play an important role in promoting actions to reduce the risks of disasters and adaptation to the impacts of climate change. Ultimately, this will help bridge the gaps that exist between the institutions that are responsible for ensuring FS. In policy areas where the interlinkages between DRR-CCA, DRR-FS, and CCA-FS are visible and the policy content is precise, KII findings showed that there is coordination, sharing of resources, clarity on mandates, roles and responsibilities, and collective use of methods, tools and strategies. This assists in reducing duplication and efficient use of resources. This article further submits that bringing coherence between the three policy areas will require officials with knowledge and understanding of the interlinkages to influence the policy processes during the review period. The major limitation of the study is that interviews were conducted with officials from government departments and research institutions funded by government only, thus neglecting cross-cutting agencies such as civil societies, private sectors, business and industry. Another limitation is the focus on national level policy and legislative frameworks only. It is also likely that these kinds of policy incoherence also manifest at the provincial and local level. Further areas of research could focus on the efficacy of institutional mechanisms needed to promote coherence between CCA, DRR and FS policies in South Africa.
